# Comparative Study of Outcomes among Patients with Polycystic Kidney Disease on Hemodialysis and Peritoneal Dialysis

**DOI:** 10.1038/srep12816

**Published:** 2015-08-06

**Authors:** Ju-Yeh Yang, Likwang Chen, Chia-Ter Chao, Yu-Sen Peng, Chih-Kang Chiang, Tze-Wah Kao, Kuo-Liong Chien, Hon-Yen Wu, Jenq-Wen Huang, Kuan-Yu Hung

**Affiliations:** 1 Division of Nephrology, Far Eastern Memorial Hospital, New Taipei City, Taiwan; 2 Institute of Population Health Sciences, National Health Research Institutes, Zhunan, Taiwan; 3 Division of Nephrology, Department of Internal Medicine, National Taiwan University Hospital, Taipei, Taiwan; 4 Department of Internal Medicine and Cardiovascular Center, National Taiwan University Hospital,Taipei, Taiwan; 5 Institute of Epidemiology and Preventive Medicine, College of Public Health, National Taiwan University, Taipei, Taiwan; 6 Health Data Research Center, National Taiwan University, Taipei, Taiwan

## Abstract

Polycystic kidney disease (PCKD) is the most common hereditary cause of end-stage renal disease, the complications of which may prevent the choice of peritoneal dialysis (PD). The aim of this study was to explore the effects of dialysis modality on outcomes in patients with PCKD. We extracted a cohort of 1417 adult patients with PCKD initiating long-term dialysis therapy in 1999–2010 from the Taiwan National Health Insurance Research Database, among which 125 patients chose PD. The patients on HD were older and had a higher comorbidity index compared to those on PD. We compared the risks for death, hospitalization and medical expenditures between the patients on PD and propensity-score matched patients on hemodialysis (HD). The overall survival did not differ between the patients on PD and HD. The patients on PD tended to have higher hazard ratios (HR) for the first episode of hospitalization (adjusted HR 1.34 [95% CI, 1.04−1.79]). The annual medical expenses were 10% lower for the patients on PD. PD is an equivalent choice of renal replacement therapy to HD for patients with PCKD in terms of survival. Although the patients on PD had a higher risk for hospitalization, the medical expenditure for PD was 10% lower.

Polycystic kidney disease (PCKD) is the most common hereditary cause of end-stage renal disease (ESRD), affecting around 500,000 persons in the United States and 4 to 6 million worldwide^1^. As the kidney cysts grow, 45% of patients will progress to ESRD by the age of 60 and up to 75% by the age of 70^2^. PCKD accounts for 8 to 10% of ESRD patients in Western countries^3^,^4^, and costs for its treatment have been reported to exceed 1.5 billion euro a year^4^. Despite multiple extra-renal complications, patients with PCKD have more favorable outcomes after ESRD compared with their counterparts with other causes of kidney failure^5^,^6^.

Based on ethical considerations, it is not possible to conduct a randomized controlled study to compare outcomes between patients on peritoneal dialysis (PD) and hemodialysis (HD), thus, there is a lack of convincing evidence on which dialysis modality is better for patients with ESRD. In most western countries, patients with ESRD were under free will to choose dialysis modality. The complications of PCKD such as enlarged kidneys and liver, diverticulosis, and the development of hernias or vascular aneurysms may theoretically prevent the choice of PD among patients with ESRD and PCKD. However, the technical survivals, quality of dialysis, duration of therapy and rates of complications for PD have been reported to be comparable in both patients with and without PCKD^7^,^8^,^9^. In addition, PD has been reported to be more frequently used and to be associated with a significantly better adjusted survival in patients with ESRD and PCKD than in those with other causes of ESRD in the United States^10^,^11^. However, evidence about direct comparisons of outcomes between patients with PCKD on PD and HD is still lacking.

The clinical diagnosis for PCKD usually relies on examination of family history and imaging studies. As PCKD is a relatively rare disease with a slow progression, it is expected that a study on this disease in a single center would only be able to enroll a small number of patients^6^,^8^,^9^. Using healthcare administrative data to conduct research on PCKD would therefore be a preferable, economical approach, which could also provide highly reliable information on the prognosis and treatment effects regarding PCKD^5^,^10^,^11^,^12^. Using this rationale, we investigated the outcomes of patients with ESRD and PCKD using the National Health Insurance Research Database (NHIRD) of Taiwan. We compared the overall survival, hospitalization risk and medical expenditure between patients with PCKD on PD and HD.

## Results

**Figure 1:**
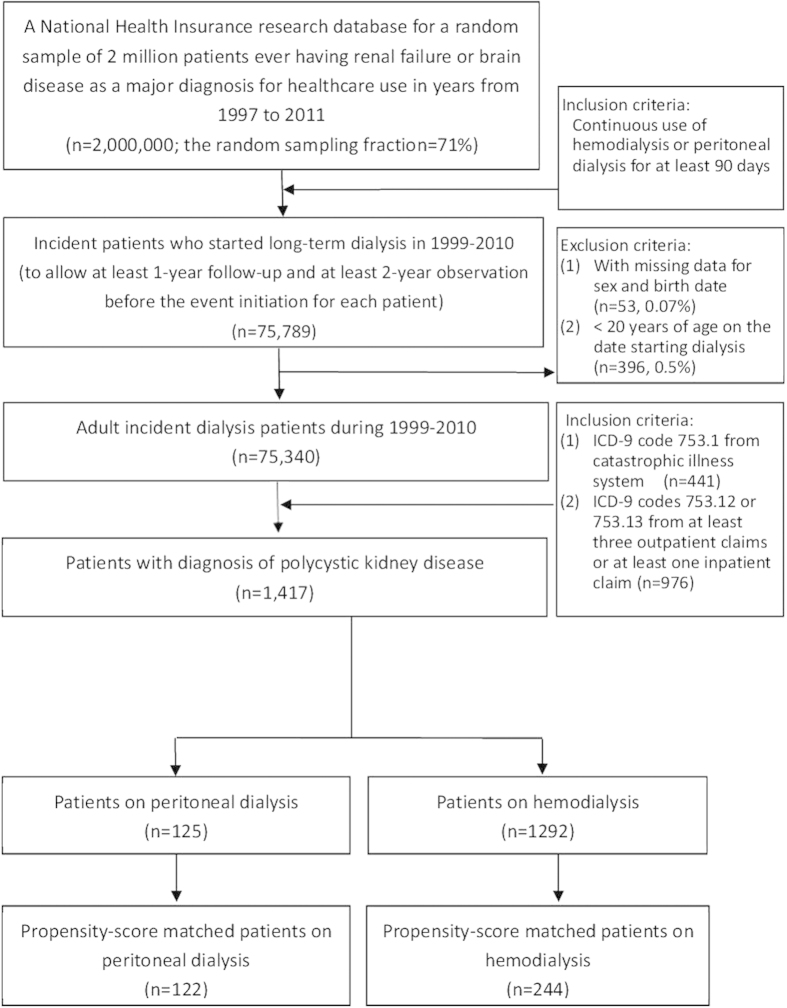
Flowchart of patient selection.

The patient selection flowchart is shown in Fig. 1. The prevalence of PCKD among the patients with ESRD in Taiwan is around 1.9%. We identified1417 patients with PCKD who initiated long-term dialysis during 1999–2010, among whom125 (8.8%) received PD. The patients on HD were older and had a higher comorbidity index compared to the patients on PD (Table 1). The median follow-up period was 3.6 years. The 1- and 5-year mortality rates after ESRD among the patients with PCKD were 8.1% and 25.2%,respectively. There was an increasing trend regarding the incident age of ESRD(mean age 55.0 in 1999 and 59.5 in 2010; p value for trend: 0.01) and the percentage of patients choosing PD as the dialysis modality among the patients with PCKD (4.2% in 1999 and 10.4% in 2010, p value for trend: 0.02)during 1999 to 2010.

**Figure 2:**
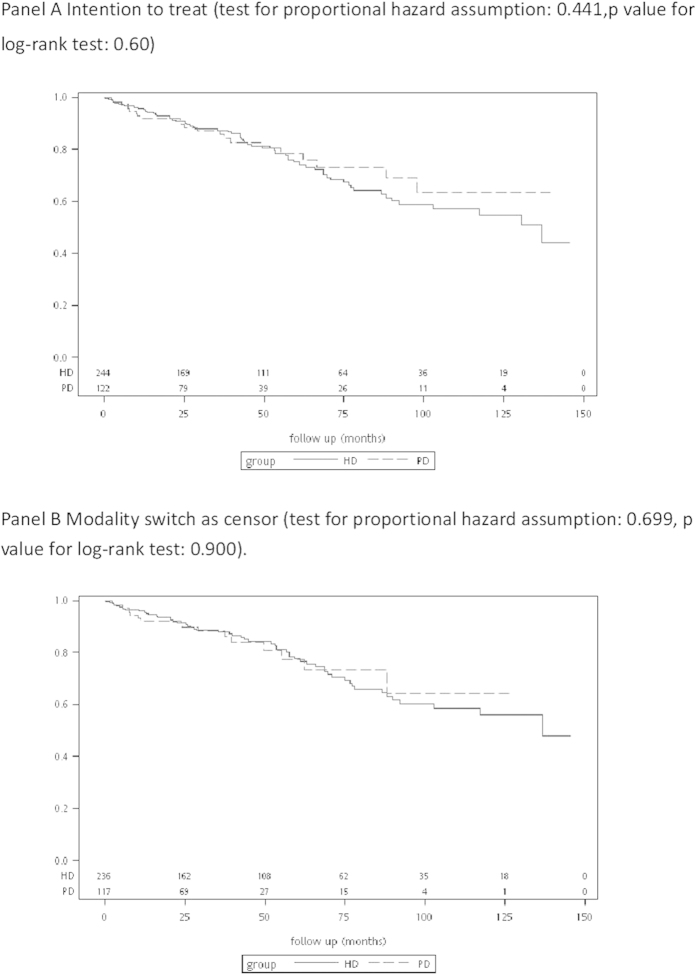
Survival curves between the patients with polycystic kidney disease on peritoneal dialysis and hemodialysis.

The characteristics of the PS-matched patients with PCKD on PD and HD are shown in Table 2. The overall survival of the patients with PCKD on PD were better compared with those on HD among the total PCKD population before adjustments (data not shown). However, in the PS-matched analysis, the overall survival rates did not differ between the patients on PD and HD, regardless of whether or not a switch in treatment modality was censored (Fig. 2).

**Figure 3:**
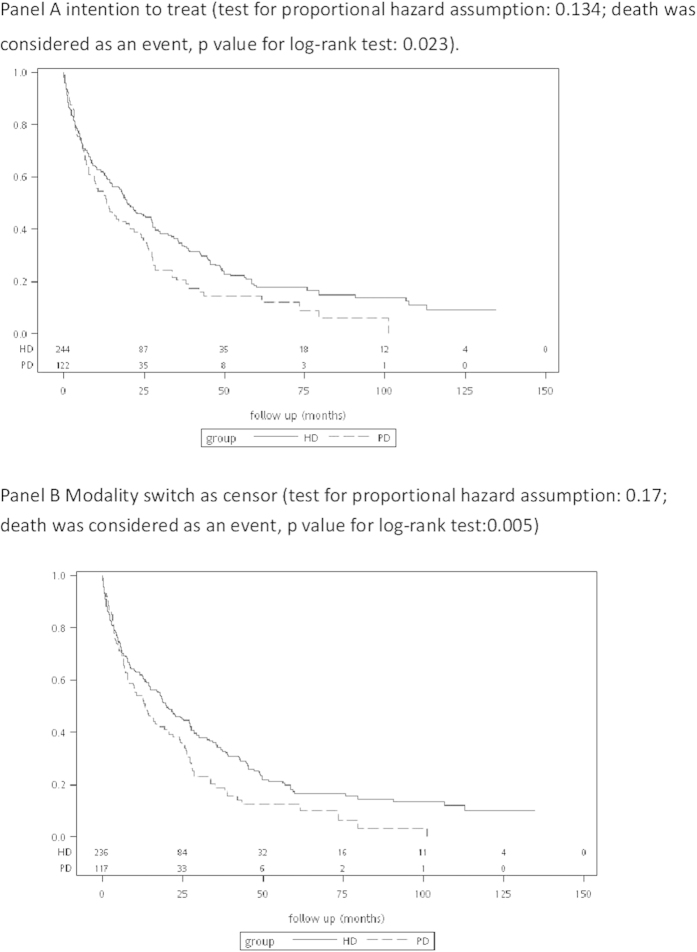
Survival curves regarding the incidence of a first episode of hospitalization among the patients with polycystic kidney disease on peritoneal dialysis and hemodialysis.

Around 70% of the patients with PCKD on dialysis experienced at least one hospitalization during the follow-up period (Table 3). The patients on PD had around 35% higher risk for the first episode of hospitalization (Fig. 3). However, after taking repeated hospitalizations into consideration, the rates of hospitalization were not significantly different between the patients undergoing PD and HD based on intention to treat analysis (Table 3). The number of hospitalizations with intensive care unit stay, length of hospital stay, and in-hospital mortality rate were similar between the patients on PD and HD. The patients on PD had a higher rate of infection-related hospitalizations(58.2% vs. 44.7%, p = 0.02, based on intention to treat analysis).

The medical expenses were significantly lower for the patients on PD (Table 4). The median annual cost of PD per patient was approximately NT$610,100, and the annual cost of HD wasapproximatelyNT$681,300. After excluding healthcare costs in the last 3 months of life, the annual expenditure of PD remained 10% lower than that for HD. The difference mainly came from outpatient care costs.

**Figure 4:**
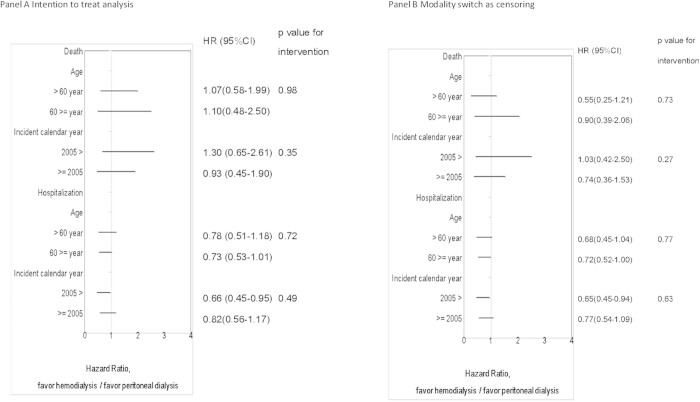
Subgroup analysis by age and incident year.

With regards to subgroup analysis (Fig. 4), we did not find and significant interactions between age or incident calendar year and modality for risk of death or hospitalization. The frequency of hernia requiring surgical intervention, subarachnoid hemrrhage or non-dialysis outpatients visits did not differ (Table 5).

## Discussion

There are numerous studies addressing survival after renal replacement therapy in patients with PCKD^4^,^6^,^8^,^9^,^10^,^11^, however, the observation of outcomes between dialysis modalities were unavoidably subject to selection bias. This is the first study aimed to compare survival, hospitalization risk and medical expenditure of patients with PCKD on PD and HD. We found comparable risks for death after adjustments for selection bias between the patients on PD and HD. Despite a slightly higher risk for hospitalizations among the patients on PD, the overall medical expenses were over 10% lower for the patients on PD even after PS-matched comparisons.

The theoretical concerns over intra-abdominal complications of PCKD did not appear to prevent patients choosing PD as the dialysis modality; on the contrary, the proportion of patients with PCKD choosing PD was higher than those without PCKD^13^. In this cohort, the timing of pre-dialysis nephrologist referral and economic status were not significant factors when choosing the modality in the PCKD cohort. Healthier and younger patients with PCKD tended to choose PD, whereas those with hypertension, heart disease, or cerebral vascular disease were less likely to select PD (Table 1). Therefore, it is not surprising that the statistical results without adequately adjusting for self-selection showed a better outcome and higher chance of kidney transplantation for the PD patients^3^,^4^,^6^,^10^,^11^. In PS-matched analysis, the patients with PCKD on HD did not have a higher risk of overall mortality, regardless of the age group or incident era of dialysis.

Hospitalization rate is an important indicator for economic burden and quality of life. Previous comparisons in patients with ESRD^14^,^15^,^16^ reported that patients on PD are at a higher risk of hospitalization than patients on HD, and especially infection-related hospitalizations^16^. This difference has been decreasing due to an increasing rate of infection-related hospitalizations among patients on HD^13^. In concordance with the observation from general ESRD population, there is also a slightly higher risk for infection related hospitalization among the patients on PD (Table 3) and an insignificant trend to favor PD after 2005 for risk of hospitalization in this PCKD cohort (Fig. 4). Probably due to the relatively small number of cases, we could not conclude whether the relative risk for hospitalization between the patients on PD and HD varied over time from the subgroup analysis. As for the severity of hospitalization, indicated by hospitalization with intensive care unit stay, length of stay and in-hospital mortality, there was no obvious difference between the patients on PD and HD.

Despite the marginally higher risk of hospitalization for the patients on PD, the overall medical expenditure of the patients on PD was 10% lower than that for those on HD. Although the costs are based on payment schedule in Taiwan, the finding is consistent with previous cost analysis between patients on PD and HD among other ESRD populations^17^,^18^,^19^. The differences mainly stemmed from cheaper outpatient care for the patients on PD and this persisted within PS-matched comparison regardless of whether or not the healthcare costs in the last 3 months of life were taken into account. Although interesting, how PCKD impacts the medical costs of patients on dialysis is beyond the scope of this study.

There are several limitations to this study. As mentioned, an observational cohort cannot completely avoid the concern of selection bias. Although we applied a PS-matched method to deal with the selection bias, we could not find a comparable match for 5 (5%)patients on PD. It would raise concerns about the generalization of the results and insufficiency of power. In addition, patients on either modality is not the same as "patient choice" of the modality. For example, there are potentially many patients that chose PD initially and could not continue due to limitations with catheter placement. Other concerns with administrative claims data include reasonable doubts about the definition of cases and events, a lack of laboratory data such as sonography reports, dialysis adequacy and direct measurements of quality of life. Furthermore, the conclusions are based on practice patterns in Taiwan and which might vary between healthcare systems.

## Methods

### Data source and quality

The NHIRD contains de-identified registration files and origin claim data for reimbursements from the Taiwan NHI program. For a project on diseases in kidneys and the brain, the National Health Research Institutes (NHRI) of Taiwan have used the NHIRD to construct a cohort of two million patients who ever used dialysis care, or ever had major diagnoses on chronic kidney disease (CKD) , acute kidney injury (AKI) or severe neurological diseases between 1997 and 2011. To comply with an NHIA policy that regulates the maximal proportion of data extracted from the population data, the NHRI set the number of patients at 2 million. The cohort is a random sample from all individuals having aforementioned conditions or diagnoses, and the sampling fraction was as high as 71%. Each patient’s longitudinal registration and claims data for 1997-2011 were collected.

The quality of NHI data has been recognized, and the data have been used in many projects on clinical epidemiology and health services research^20^. The reliability of NHI data has been demonstrated in a body of literature on validation of specific disease diagnoses in the NHI data^21^,^22^,^23^. Because the NHIA has an auditing system for preventing fraud, the quality of NHI records is also guaranteed in regard to expensive procedures and health services, as well as medications^24^. The NHIA also operates an auditing system for the NHI catastrophic illness registry system, because enrollees in this special system receive additional benefits in healthcare.

### Study population

On the basis of the cohort with 2-million patients, we further constructed a sample for the study. Adult patients (>=20 years old) who initiated maintenance dialysis for more than 3 months during 1999 to 2010 were included, to allow at least 1year of observation after the event and at least 2years before the event for each patient. Individuals who received PD 2 to 4 months after the initiation of dialysis were defined as the PD cohort, regardless of whether they changed the modality at a later date.

We identified patients with PCKD registered with a catastrophic illness with an ICD-9-CM diagnostic code of 753.1X (cystic kidney disease). Patients with PCKD are eligible to register with the catastrophic illness registry in Taiwan, however registration is not required. Therefore, we also defined patients with PCKD by specific ICD-9-CM codes 753.12 (polycystic kidney, unspecified type) or 753.13 (polycystic kidney, autosomal dominant) from at least three outpatient claims or at least one inpatient claim during 1997–2011.

### Study outcomes

The primary outcome of interest was mortality. The date of death was defined from records in the registry of catastrophic illnesses or the discharge status (death or critically ill discharge). Patients were defined as being lost to follow-up if there were no registry records or claims records for more than 1 year.

The secondary outcomes included risk for hospitalization of any cause, incidence of subarachnoid hemorrhage and abdominal hernia requiring surgical intervention. We further examined the severity of hospitalization as indicated by the length of stay, intensive care unit stay, in-hospital mortality, and infection-related hospitalization, in particular abdominal infections.

We calculated the average annual expenditure of each patient after the initiation of dialysis, excluding the healthcare costs in the last 3 months of life and extracting expenses for inpatient and outpatient care. We calculated the expenditure according to the payment schedule of the NHI global budgeting system based on the Consumer Price Index of Taiwan. All expenditures were expressed in Taiwan dollars based on the value in31 December 2013 when the exchange rate to one US dollar was 29.997.

### Candidate predictors

In addition to age and sex, we recorded the socioeconomic status from the registry data within 6 months of the initiation of PD. Socioeconomic status was classified into three groups according to insurance premium levels.

We ascertained 12 comorbidities from both inpatient and outpatient claims of the 2 years before the initiation of dialysis, including diabetes, heart failure, coronary artery disease, chronic obstructive pulmonary disease, cerebral vascular disease, hypertension, arrhythmia, valvular heart disease, peripheral vascular disease, chronic liver disease, dyslipidemia, and gout. We calculated Deyo’s Charlson comorbidity index^25^ according to claims data 2 years before the initiation of dialysis. Late referral to a nephrologist referred to a duration between the first nephrologist visit to dialysis of less than 180 days.

### Statistical analysis

We used the Student’s t-test and Wilcoxon signed-rank test to compare continuous variables, and the chi-square test or Fisher’s exact test and Cochran–Mantel–Haenszel (CMH) test for categorical variables, accordingly. For expense data, we used logarithmic transformations before comparisons. Since the dialysis modality was not randomly assigned, we performed propensity-score (PS) matching analysis to adjust for self-selection. The logistic regression model to calculate the PS and some diagnostic analyses were shown in Appendix. We performed the analysis both based on intention to treat (regardless of whether the dialysis modality was switched or not during the follow-up period) and considering modality switch as censoring.

We used Cox regression models to evaluate the associations among potential risk factors and event free survival. We reported logit PS adjusted risk ratios (HR) and 95% confidence intervals (CI) for each factor. For analyses based on intention to treat, patients were censored after successful renal transplantation, if they were lost to follow-up, or on December 31, 2010. We performed sensitivity analyses by additionally censoring the modality switch. We grasped the interval when the patients were temporary on another dialysis modality for less than 60 days. Only those patients who shifted to another modality for more than 60 days were censored. We also performed sensitivity analyses considering renal transplantation as a competing event, which did not materially changed the results (see Appendix).

Considering hospitalizations as a recurrent outcome, we used a negative binomial model to estimate the relative risk for hospitalization adjusted to the length of time for observation.

We performed subgroup analysis by age and calendar year to evaluate potential effect modifiers between modalities and outcomes. We divided the population before and after 2005 as icodextrin was covered by the NHI program in Taiwan in 2005.

All analyses were performed using SAS software version 9.3 (The SAS Institute Inc., Cary, NC).

This study protocol was approved by the Institutional Review Board of National Taiwan University Hospital. The methods were carried out in accordance with the approved guidelines. Informed consent was waived because the dataset is encrypted and de-identified.

## Conclusions

In conclusion, the cohort in this study is the largest published cohort of Asian patients with PCKD on dialysis with longitudinal follow-up over 12 years from a reliable national dataset. PD was an equivalent choice of renal replacement therapy to HD for the patients with PCKD in terms of overall survival. Although the patients with PCKD on PD had a slightly higher risk for hospitalization, the medical expenditure of the patients on PD was 10% lower based on the payment schedule in Taiwan.

## Additional Information

**How to cite this article**: Yang, J.-Y. *et al.* Comparative Study of Outcomes among Patients with Polycystic Kidney Disease on Hemodialysis and Peritoneal Dialysis. *Sci. Rep.* **5**, 12816; doi: 10.1038/srep12816 (2015).

## Supplementary Material

Supplementary Information

## Figures and Tables

**Table 1:** Baseline characteristics of incident long-term dialysis (>90 days) adult patients with polycystic kidney disease (>=20 years) during 1999–2010.

	PD(n = 125)	HD(n = 1292)	P value
Age, years, mean (std)	53.6(14.9)	60.3(13.9)	<0.001
Male, n (%)	62(50.4)	760(58.8)	0.068
Comorbidity index, mean (std)	2.79(1.14)	3.4(1.72)	<0.001
Late referral to a nephrologist, n (%)	17(13.6)	234(18.2)	0.200
Economic status			
Low	41(32.8)	442(34.2)	0.364
Intermediate	76(60.8)	801(62.0)	
High	8(6.4)	49(3.8)	
Comorbidity			
Diabetes	15(12.0)	233(18.0)	0.09
Hypertension	87(69.6)	1043(80.7)	0.003
Coronary artery disease	18(14.4)	275(21.3)	0.070
Cerebral vascular disease	4(3.2)	121(9.4)	0.019
Heart failure	12(9.6)	227(17.6)	0.023
Arrhythmia	8(6.4)	116(9.0)	0.330
Valvular heart disease	4(3.2)	82(6.4)	0.159
Peripheral vascular disease	0(0)	40(3.1)	0.044
COPD	4(3.2)	134(10.4)	0.007
Chronic liver disease	19(15.2)	185(14.3)	0.789
Gout	42(33.6)	376(29.1)	0.292
Dyslipidemia	19(15.2)	151(11.7)	0.248
Cancer	4(3.2)	93(7.2)	0.097

**Table 2:** Baseline characteristics of propensity-score matched patients with polycystic kidney disease on peritoneal dialysis and hemodialysis.

	PD(n = 122)	HD(n = 244)	P value
Age, years, mean (std)	54.0(14.7)	54.0(13.3)	0.969
Male, n (%)	62(50.8)	130(53.3)	0.657
Comorbidity index, mean (std)	2.80(1.15)	2.88(1.32)	0.534
Late referral to a nephrologist, n (%)	16(13.1)	47(19.3)	0.143
Economic status			
Low	40(32.8)	81(33.2)	0.847
Intermediate	75(61.5)	145(59.4)	
High	7(5.7)	18(7.4)	
Comorbidity			
Diabetes	15(12.3)	26(10.7)	0.640
Hypertension	86(70.5)	172(70.5)	1.00
Coronary artery disease	18(14.8)	39(16.0)	0.760
Cerebral vascular disease	4(3.3)	6(2.5)	0.651
Heart failure	12(9.8)	27(11.1)	0.720
Arrhythmia	8(6.6)	16(6.6)	1.000
Valvular heart disease	4(3.3)	12(4.9)	0.470
Peripheral vascular disease	0	0	N/A
COPD	4(3.3)	9(3.7)	0.842
Chronic liver disease	19(15.6)	33(13.5)	0.597
Gout	41(33.6)	89(36.5)	0.589
Dyslipidemia	17(13.9)	29(11.9)	0.578
cancer	4(3.3)	8(3.3)	1.0000

**Table 3:** Risks for hospitalization between patients with polycystic kidney disease on peritoneal dialysis and propensity-scored matched hemodialysis patients.

	Peritoneal dialysis	Hemodialysis	P value
Intention to treat analysis			
Hospitalization cases	92(78.0)	165(69.9)	0.110
Hospitalization rates, median (Q1–Q3), per person-year	0.7(0.2–1.6)	0.6(0.2–1.4)	0.609
HR for first episode of hospitalization^1^	1.34 (1.04–1.79)	Reference	0.026
RRR for hospitalizations^1^	1.13(0.87–1.46)	Reference	0.371
Hospitalization for infection, n (%)	71(58.2)	109(44.7)	0.015
Length of stay, median (Q1–Q3)	20.5(3.0–50.0)	15.0(5.5–41.0)	0.432
Hospitalization for abdominal infection, n (%)	11(9.0)	21(8.6)	0.896
Severe hospitalization, n (%)			
Intensive care unit stay	39(32.0)	82(33.6)	0.754
>=30 days	20(16.4)	35(14.3)	0.606
Death during hospitalization, n(%)	13(10.7)	33(13.5)	0.436
Modality switch and transplantation as censor			
Hospitalization cases	90(76.3)	165(69.9)	0.210
hospitalization rates, median (Q1–Q3), per person-year	0.7(0.2–1.7)	0.6(0.2–1.4)	0.348
Hospitalization for infection, n (%)	65(55.1)	104(44.1)	0.051
Length of stay, median (Q1–Q3)	14.5(3.0–40.0)	15.0(5.5–41.0)	0.376
Hospitalization for abdominal infection, n (%)	9(7.6)	20(8.5)	0.784
HR for first episode of hospitalization^1^	1.40(1.06–1.84)	Reference	0.017
RRR for hospitalizations^1^	1.39 (1.08–1.79)	Reference	0.011
Severe hospitalization, n (%)			
Intensive care unit stay	29(24.6)	76(32.2)	0.139
>= 30 days	15(12.7)	34(14.4)	0.664
Death during hospitalization, n(%)	10(8.5)	29(12.3)	0.281

^1^Adjusted for logit propensity-score.

**Table 4:** Medical expenditure comparison between patients with polycystic kidney disease on peritoneal dialysis and propensity-scored matched patients on hemodialysis.

Medical expense (1000 NT$/py), median (Q1–Q3)	Peritoneal dialysis (n = 122)	Hemodialysis (n = 244)	P value
Intention to treat			
Total	610.1(471.2–706.3)	681.3(624.9–754.2)	<0..001
Excluding the healthcare costs in the last 3 months of life	612.5(471.0–716.9)	680.1(618.3–756.4)	<.0001
Cost for hospitalization	41.8(4.0–113.2)	20.6(0–95.2)	0.854
Cost for outpatient	519.5(451.8–597.0)	639.5(584.1–686.5)	<0.001
Modality switch as censoring			
Total	557.4(165.6–655.3)	681.3(623.9–753.9)	<0.001
Excluding the healthcare costs in the last 3 months of life	564.1(462.8–703.0)	678.7(617.8–752.7)	<0.001
Cost for hospitalization	31.5(1.3–100.4)	20.6(0–95.2)	0.677
Cost for outpatient	511.6(432.9–582.1)	639.5(582.8–686.8)	<0.001

**Table 5:** Other outcome comparisons between patients with polycystic kidney disease on peritoneal dialysis and propensity-scored matched patients on hemodialysis.

	peritoneal dialysis (n = 122)	hemodialysis (n = 244)	P value
Transplantations	14(11.5)	24(9.8)	0.623
Modality switch	31(25.4)	3(1.2)	<0.001
Intention to treat analysis			
Hernia requiring surgical intervention	8(6.6)	10(4.1)	0.306
Death cases, n (%)	22(18.0)	62(25.4)	0.114
Subarachnoid hemorrhage cases	3(2.5)	1(0.4)	0.076
Rate for non-dialysis outpatient visits, median (Q1–Q3), per person-year	18.4(12.7–28.9)	17.3(10.4–28.2)	0.813
Modality switch as censoring			
Hernia requiring surgical intervention	7(5.9)	10(4.2)	0.483
Death cases, n (%)	17(14.4)	55(23.3)	0.050
Subarachnoid hemorrhage cases	2(1.7)	1(0.4)	0.219
Rate for non-dialysis outpatient visits, median (Q1–Q3), per person-year	17.4(11.1–29.5)	17.3(10.3–28.2)	0.813

## References

[b1] Wilson P. D. Polycystic kidney disease. The New England journal of medicine 350, 151–164 (2004). 14711914 10.1056/NEJMra022161

[b2] Gabow P. A. *et al.* Factors affecting the progression of renal disease in autosomal-dominant polycystic kidney disease. Kidney international 41, 1311–1319 (1992). 1614046 10.1038/ki.1992.195

[b3] Perrone R. D., Ruthazer R. & Terrin N. C. Survival after end-stage renal disease in autosomal dominant polycystic kidney disease: contribution of extrarenal complications to mortality. American journal of kidney diseases : the official journal of the National Kidney Foundation 38, 777–784 (2001). 11576881 10.1053/ajkd.2001.27720

[b4] Spithoven E. M. *et al.* Renal replacement therapy for autosomal dominant polycystic kidney disease (ADPKD) in Europe: prevalence and survival—an analysis of data from the ERA-EDTA Registry. Nephrology, dialysis, transplantation : official publication of the European Dialysis and Transplant Association - European Renal Association 29 Suppl 4, iv15–iv25 (2014). 10.1093/ndt/gfu017 PMC7611099 25165182

[b5] van Walraven C., Manuel D. G. & Knoll G. Survival trends in ESRD patients compared with the general population in the United States. American journal of kidney diseases : the official journal of the National Kidney Foundation 63, 491–499 (2014). 24210591 10.1053/j.ajkd.2013.09.011

[b6] Haynes R., Kheradmand F. & Winearls C. G. Survival after starting renal replacement treatment in patients with autosomal dominant polycystic kidney disease: a single-centre 40-year study. Nephron. Clinical practice 120, c42–c47 (2012). 22205054 10.1159/000334429

[b7] Prischl F. C. *et al.* Peritoneal dialysis in patients with polycystic kidney disease. Wiener klinische Wochenschrift 117 Suppl 6, 24–28 (2005). 16437329 10.1007/s00508-005-0492-y

[b8] Li L. *et al.* Peritoneal dialysis as the first-line renal replacement therapy in patients with autosomal dominant polycystic kidney disease. American journal of kidney diseases : the official journal of the National Kidney Foundation 57, 903–907 (2011). 21458901 10.1053/j.ajkd.2011.01.019

[b9] Kumar S., Fan S. L., Raftery M. J. & Yaqoob M. M. Long term outcome of patients with autosomal dominant polycystic kidney diseases receiving peritoneal dialysis. Kidney international 74, 946–951 (2008). 18650794 10.1038/ki.2008.352

[b10] Abbott K. C. & Agodoa L. Y. Polycystic kidney disease at end-stage renal disease in the United States: patient characteristics and survival. Clinical nephrology 57, 208–214 (2002). 11924752 10.5414/cnp57208

[b11] Reule S. *et al.* ESRD from autosomal dominant polycystic kidney disease in the United States, 2001-2010. American journal of kidney diseases : the official journal of the National Kidney Foundation 64, 592–599 (2014). 25134777 10.1053/j.ajkd.2014.05.020 PMC4396817

[b12] Lee P. W., Chien C. C., Yang W. C., Wang J. J. & Lin C. C. Epidemiology and mortality in dialysis patients with and without polycystic kidney disease: a national study in Taiwan. Journal of nephrology 26, 755–762 (2013). 23042434 10.5301/jn.5000224

[b13] Collins A. J. *et al.* US Renal Data System 2013 Annual Data Report. American journal of kidney diseases : the official journal of the National Kidney Foundation 63, A7 (2014). 24360288 10.1053/j.ajkd.2013.11.001

[b14] Murphy S. W. *et al.* Comparative hospitalization of hemodialysis and peritoneal dialysis patients in Canada. Kidney international 57, 2557–2563 (2000). 10844625 10.1046/j.1523-1755.2000.00115.x

[b15] Lafrance J. P. *et al.* Association of dialysis modality with risk for infection-related hospitalization: a propensity score-matched cohort analysis. Clinical journal of the American Society of Nephrology : CJASN 7, 1598–1605 (2012). 22904124 10.2215/CJN.00440112 PMC3463202

[b16] Kumar V. A., Ledezma M. L., Idroos M. L., Burchette R. J. & Rasgon S. A. Hospitalization rates in daily home hemodialysis versus peritoneal dialysis patients in the United States. American journal of kidney diseases : the official journal of the National Kidney Foundation 52, 737–744 (2008). 18752877 10.1053/j.ajkd.2008.06.013

[b17] Shih Y. C., Guo A., Just P. M. & Mujais S. Impact of initial dialysis modality and modality switches on Medicare expenditures of end-stage renal disease patients. Kidney international 68, 319–329 (2005). 15954923 10.1111/j.1523-1755.2005.00413.x

[b18] Lee H. *et al.* Cost analysis of ongoing care of patients with end-stage renal disease: the impact of dialysis modality and dialysis access. American journal of kidney diseases : the official journal of the National Kidney Foundation 40, 611–622 (2002). 12200814 10.1053/ajkd.2002.34924

[b19] Kao T. W. *et al.* Lifetime costs for peritoneal dialysis and hemodialysis in patients in Taiwan. Peritoneal dialysis international : journal of the International Society for Peritoneal Dialysis 33, 671–678 (2013). 23636434 10.3747/pdi.2012.00081 PMC3862097

[b20] Yeh H.-Y., Wu J.-C., Haschler I., Chen T.-J. & Wetter T. Taiwan's National Health Insurance Research Database: administrative health care database as study object in bibliometrics. Scientometrics 86, 365–380 (2011).

[b21] Wu V. C. *et al.* Administrative data on diagnosis and mineralocorticoid receptor antagonist prescription identified patients with primary aldosteronism in Taiwan. Journal of clinical epidemiology 67, 1139–1149 (2014). 25034196 10.1016/j.jclinepi.2014.05.012

[b22] Lin C. C., Lai M. S., Syu C. Y., Chang S. C. & Tseng F. Y. Accuracy of diabetes diagnosis in health insurance claims data in Taiwan. Journal of the Formosan Medical Association = Taiwan yi zhi 104, 157–163 (2005). 15818428

[b23] Cheng C. L., Kao Y. H., Lin S. J., Lee C. H. & Lai M. L. Validation of the National Health Insurance Research Database with ischemic stroke cases in Taiwan. Pharmacoepidemiology and drug safety 20, 236–242 (2011). 21351304 10.1002/pds.2087

[b24] Wu V. C. *et al.* Long-term risk of coronary events after AKI. Journal of the American Society of Nephrology : JASN 25, 595–605 (2014). 24503241 10.1681/ASN.2013060610 PMC3935592

[b25] Quan H. *et al.* Coding algorithms for defining comorbidities in ICD-9-CM and ICD-10 administrative data. Medical care 43, 1130–1139 (2005). 16224307 10.1097/01.mlr.0000182534.19832.83

