# Supplementary material for: Comparative Study of Outcomes among Patients with Polycystic Kidney Disease on Hemodialysis and Peritoneal Dialysis
Source: Sci Rep. 2015 Aug 6;5:12816. doi: 10.1038/srep12816 (PMC4526846; doi:10.1038/srep12816)

Comparative Study of Outcomes among Patients with Polycystic Kidney Disease on Hemodialysis and Peritoneal Dialysis

Ju-Yeh Yang [1], Likwang Chen[2], Chia-Ter Chao[3], Yu-Sen Peng[1], Chih-Kang Chiang[3], Tze-Wah Kao[3], Kuo-Liong Chien[4,5,6], Hon-Yen Wu[1,5], Jenq-Wen Huang *[3], Kuan-Yu Hung[3]

Appendix

Appendix 1. The logistic regression model to calculate the probability of receiving PD.

Parameter		DF	Estimate	Standard Error	Wald Chi-Square	Pr > ChiSq
Intercept		1	-0.0493	0.5784	0.0073	0.9320
ID_SEX	F	1	0.4885	0.2007	5.9249	0.0149
Age		1	-0.0289	0.00793	13.2613	0.0003
economic	0	1	-0.5228	0.4287	1.4876	0.2226
economic	1	1	-0.6075	0.4111	2.1842	0.1394
DM	1	1	-0.2822	0.3030	0.8677	0.3516
HF	1	1	-0.1423	0.3431	0.1721	0.6782
CAD	1	1	-0.0396	0.2929	0.0182	0.8926
COPD	1	1	-0.7893	0.5293	2.2237	0.1359
CVD	1	1	-0.8755	0.5272	2.7577	0.0968
Cancer	1	1	-0.6796	0.5291	1.6497	0.1990
HTN	1	1	-0.4355	0.2205	3.9003	0.0483
Arrhy	1	1	0.0834	0.4059	0.0423	0.8371
VHD	1	1	-0.3148	0.5431	0.3360	0.5621
PVD	1	1	-13.7222	561.8	0.0006	0.9805
Liver	1	1	0.1362	0.2695	0.2552	0.6134
Lipid	1	1	0.4844	0.2823	2.9439	0.0862
Gout	1	1	0.4971	0.2155	5.3196	0.0211

Appendix 2. Standardized differences in the unmatched opulation

	Mean in treated	Mean in Untreated	Standardised diff.
Age	53.56	60.27	-0.466
sex	0.50	0.59	-0.169
economic	0.74	0.70	0.073
dm	0.12	0.18	-0.169
hf	0.10	0.18	-0.234
cad	0.14	0.21	-0.180
copd	0.03	0.10	-0.288
cvd	0.03	0.09	-0.256
Cancer	0.03	0.07	-0.181
htn	0.70	0.81	-0.259
Arrhy	0.06	0.09	-0.097
vhd	0.03	0.06	-0.148
pvd	0.00	0.03	-0.253
Liver	0.15	0.14	0.025
Lipid	0.15	0.12	0.103
Gout	0.34	0.29	0.097

Appendix 3.Standardized differences in the matched population

	Mean in treated	Mean in Untreated	Standardised diff.
Age	53.97	54.01	-0.003
sex	0.51	0.53	-0.049
economic	0.73	0.74	-0.021
dm	0.12	0.11	0.051
hf	0.10	0.11	-0.040
cad	0.15	0.16	-0.034
copd	0.03	0.04	-0.022
cvd	0.03	0.02	0.049
Cancer	0.03	0.03	0.000
htn	0.70	0.70	-0.000
Arrhy	0.07	0.07	0.000
vhd	0.03	0.05	-0.083
pvd	0.00	0.00	.
Liver	0.16	0.14	0.058
Lipid	0.14	0.12	0.061
Gout	0.34	0.36	-0.060

Appendix 4. The ROC curve for the logistic regression model to calculate propensity score.

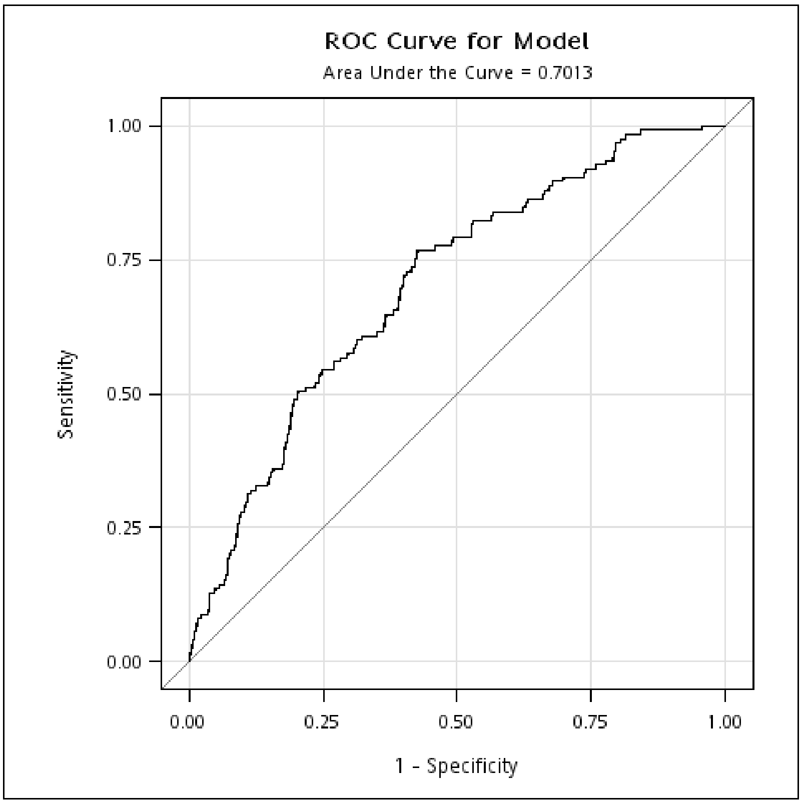

Appendix 5. Survival curves between the patients with polycystic kidney disease on peritoneal dialysis and hemodialysis, considering renal transplantation as a competing event

Panel A Intention to treat (Substantial hazard ratio for death: 0.86 (0.49-1.52), p=0.6))

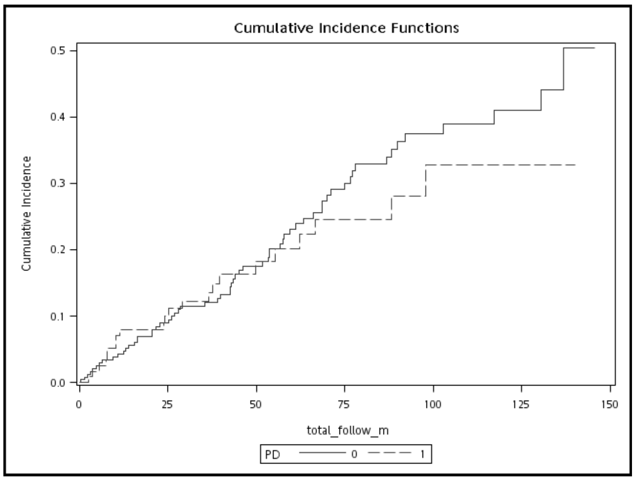

Panel B Modality switch as censor (Substantial hazard ratio for death: 0.98 (0.53-1.80), p=0.94))

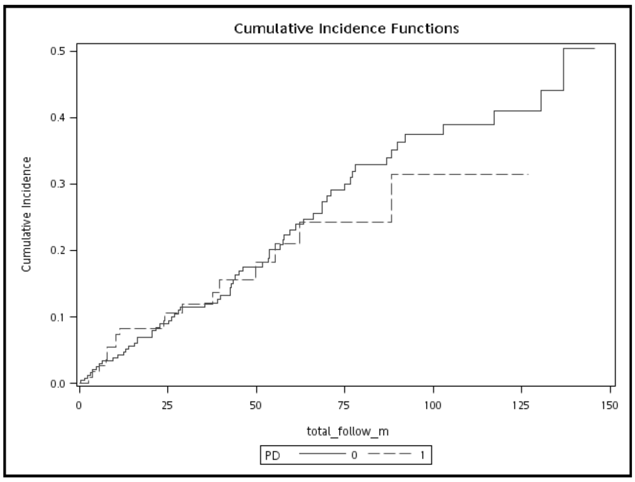

Appendix 6. Survival curves regarding the incidence of a first episode of hospitalization among the patients with polycystic kidney disease on peritoneal dialysis and hemodialysis, considering renal transplantation as a competing event

Panel A Intention to treat (Substantial hazard ratio for hospitalization: 1.46 (1.14-1.89), p=0.003))

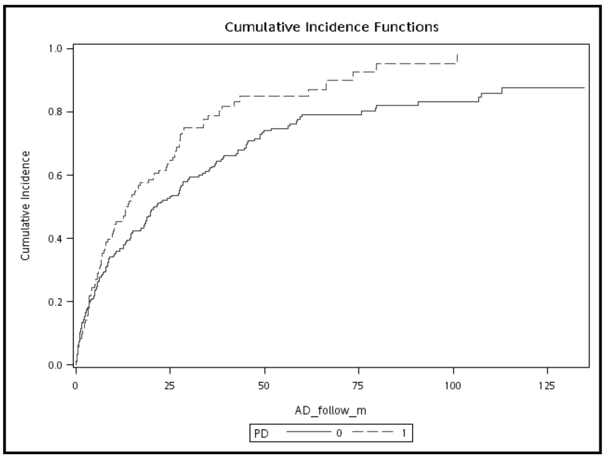

Panel B Modality switch as censor (Substantial hazard ratio for death: 1.42 (1.10-1.83), p=0.008))

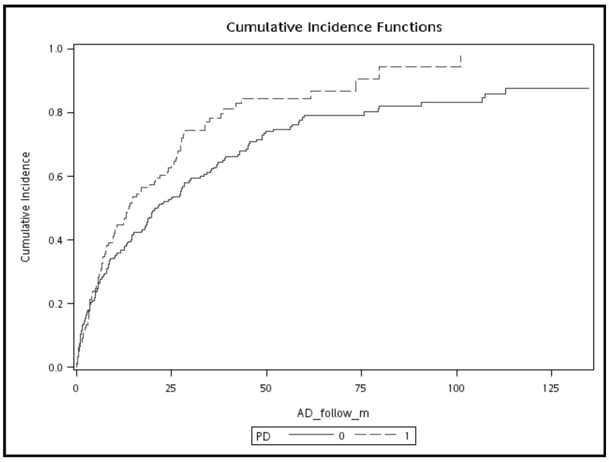

Supplement: Supplementary Information [file srep12816-s1.pdf]
